# Polypeptide Modulators of TRPV1 Produce Analgesia without Hyperthermia

**DOI:** 10.3390/md11125100

**Published:** 2013-12-16

**Authors:** Yaroslav A. Andreev, Sergey A. Kozlov, Yuliya V. Korolkova, Igor A. Dyachenko, Dmitrii A. Bondarenko, Denis I. Skobtsov, Arkadii N. Murashev, Polina D. Kotova, Olga A. Rogachevskaja, Natalia V. Kabanova, Stanislav S. Kolesnikov, Eugene V. Grishin

**Affiliations:** 1 Shemyakin-Ovchinnikov Institute of Bioorganic Chemistry, Russian Academy of Sciences, 16/10 Miklukho-Maklaya Str., Moscow 117997, Russia; E-Mails: shifter2007@gmail.com (Y.A.A.); july@ibch.ru (Y.V.K.); grev@ibch.ru (E.V.G.); 2 Branch of Shemyakin-Ovchinnikov Institute of Bioorganic Chemistry, Russian Academy of Sciences, 6 Nauki ave., Pushchino 142290, Moscow Region, Russia; E-Mails: dyachenko@bibch.ru (I.A.D.); catameno777@gmail.com (D.A.B.); skob2000@mail.com (D.I.S.); murashev@bibch.ru (A.N.M.); 3 Pushchino State Institute of Natural Sciences, 3 Nauki ave, Pushchino 142290, Moscow Region, Russia; 4 Institute of Cell Biophysics, Russian Academy of Sciences, 3 Institutskaya Str., Pushchino 142290, Moscow Region, Russia; E-Mails: kpd-88@list.ru (P.D.K.); Olga_rog@rambler.ru (O.A.R.); kabanovatata@mail.ru (N.V.K.); staskolesnikov@yahoo.com (S.S.K.)

**Keywords:** sea anemone, analgesic polypeptide APHC, TRPV1 receptor, animal models, temperature regulation, nociception, inflammation

## Abstract

Transient receptor potential vanilloid 1 receptors (TRPV1) play a significant physiological role. The study of novel TRPV1 agonists and antagonists is essential. Here, we report on the characterization of polypeptide antagonists of TRPV1 based on *in vitro* and *in vivo* experiments. We evaluated the ability of APHC1 and APHC3 to inhibit TRPV1 using the whole-cell patch clamp approach and single cell Ca^2+^ imaging. *In vivo* tests were performed to assess the biological effects of APHC1 and APHC3 on temperature sensation, inflammation and core body temperature. In the electrophysiological study, both polypeptides partially blocked the capsaicin-induced response of TRPV1, but only APHC3 inhibited acid-induced (pH 5.5) activation of the receptor. APHC1 and APHC3 showed significant antinociceptive and analgesic activity *in vivo* at reasonable doses (0.01–0.1 mg/kg) and did not cause hyperthermia. Intravenous administration of these polypeptides prolonged hot-plate latency, blocked capsaicin- and formalin-induced behavior, reversed CFA-induced hyperalgesia and produced hypothermia. Notably, APHC3’s ability to inhibit the low pH-induced activation of TRPV1 resulted in a reduced behavioural response in the acetic acid-induced writhing test, whereas APHC1 was much less effective. The polypeptides APHC1 and APHC3 could be referred to as a new class of TRPV1 modulators that produce a significant analgesic effect without hyperthermia.

## 1. Introduction

The transient receptor potential vanilloid 1 receptor (TRPV1) is a nonselective cationic ion channel that is activated by a variety of stimuli: noxious heat (>43 °C), protons, various lipid messengers and exogenous ligands, and most notably the pungent ingredient of chili peppers—capsaicin [1,2,3]. The TPRV1 receptor is believed to play a key role in temperature sensation and may be a molecular integrator for different stimuli [1,2]. TRPV1 receptors are primarily expressed in a population of small and medium diameter peptidergic sensory neurons called nociceptors, which can release proinflammatory and pronociceptive mediators and transmit nociceptive information from peripheral tissues to the spinal cord. Activation of the TRPV1 receptor *in vivo* sends an afferent pain signal to the CNS and releases pain mediators (substance P, calcitonin gene-related peptide (CGRP) and others), which lead to the development of local neurogenic inflammation [4].

TRPV1 is involved in the progress of different pathological states such as diabetic painful neuropathy, peripheral neuropathic pain, cancer pain, rheumatoid arthritis, osteoarthritis, chronic persistent cough, fecal incontinence, pain of the urinary bladder, cystitis and inflammatory bowel disease [5]. Thus, the isolation of natural compounds and the chemical design of substances capable of modulating TRPV1 are of great interest [6].

A number of small organic molecules were found to inhibit TRPV1 selectively at nanomolar concentrations *in vitro*,and some of the molecules have significant effects *in vivo*. For instance, a small molecular weight antagonist of TRPV1 produced positive pharmacodynamic effects, an increase in the heat threshold and a decrease in capsaicin-induced flare/dermal vasodilatation, in clinical trials [7].

Unfortunately, almost all small organic TRPV1 antagonists cause significant hyperthermia in animal models and a hyperthermic effect led to the termination of clinical trials for some attractive TRPV1 antagonists [8,9,10,11,12]. However, TRPV1 antagonists that did not block all modes of receptor activation and did not cause a hyperthermic effect were found [12,13].

Polypeptide molecules typically have a greater specific activity at a particular receptor than do small molecule agonists, which makes them attractive for researchers and pharmaceutical companies. We have previously reported the isolation of a polypeptide modulator of TRPV1 from a homogenized extract of the sea anemone *Heteractis crispa* named APHC1 and its partial *in vivo* characterization [14,15]. Two homological polypeptides (APHC2 and APHC3) were subsequently found in the same extract [16], but their biological activity has not yet been properly characterized. APHC1 and APHC3 are weak inhibitors of serine proteases [14,16] and modulate TRPV1-dependent normal and diabetic bladder smooth muscle contractility [17]. Here, we characterized the inhibitory action of the polypeptide APHC3 on TRPV1 receptor. We show evidence of the amelioration of the pain response by APHC1 and APHC3 in acute and chronic rodent pain models and we show the influence of both polypeptides on core body temperature in mice.

## 2. Results and Discussion

### 2.1. Pharmacology of APHC3

**Figure 1:**
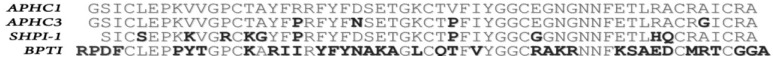
Polypeptides sequences for APHC1 and APHC3 aligned with BPTI/Kunitz type proteinase inhibitors from sea anemone *Stichodactyla helianthus*—SHPI-1 [19] and from bovine—BPTI [20,21]. Amino acids residues that differ from the APHC1 sequence are highlighted.

APHC3’s mode of action was measured on HEK-293 cells using a whole-cell patch clamp approach and single cell Ca^2+^ imaging. Because APHC1 has been partially studied in a previous study [14], we did not analyze its effects on the capsaicin-induced current of the TRPV1 receptor. APHC1 has been shown to be a partial antagonist of capsaicin-induced TRPV1 currents with EC_50_ 54 nM. APHC3 is highly homologous to APHC1, differing in four of 56 amino acids. The positions of substitutions are shown in Figure 1. APHC1 and APHC3 have a primary structure that is highly homologous to BPTI/Kunitz type serine-protease inhibitors and K^+^ channel blockers from sea anemones [18].

Several polypeptide modulators of TRPV1 that interact with the extracellular part of the receptor have been reported. Spider’s polypeptides, the vanilotoxins 1–3 and DkTx, activate TRPV1 *in vitro* and mediate the pain response when injected in mice [22,23]. In addition, known rabbit polyclonal antibodies against the pre-pore region of TRPV1 partially inhibited receptor activation *in vitro* [24]. The structural homology of APHCs to these proteins is minimal so both polypeptides presented structural distinct class of TRPV1 inhibitors.

**Figure 2:**
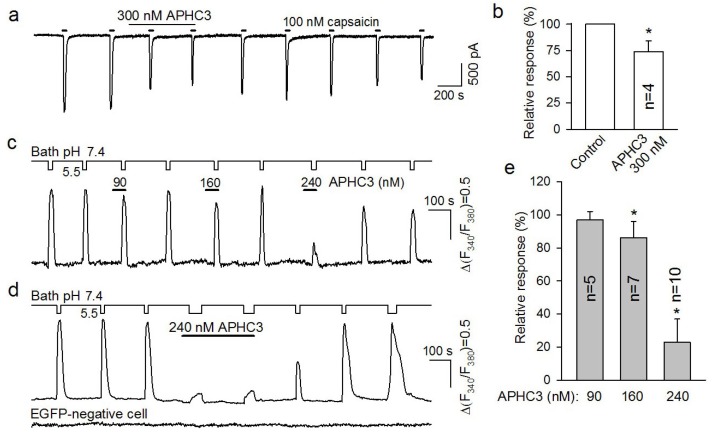
Effects of APHC3 on recombinant TRPV1 expressed in HEK-293 cells. (**a**) Resting current recorded from a TRPV1-positive cell held at −30 mV. Capsaicin (100 nM) elicited transient inward currents that were partially inhibited in the presence of the APHC3 peptide (300 nM). Straight lines above the recording indicate application of APHC3 (Long line) and capsaicin (Short lines). The cells were stimulated and rinsed by rapid switching of the bath solution between the control solution and solutions containing the indicated compound. (**b**) Capsaicin responses in control and in the presence of the 300 nM APHC3 peptide. In each case, the control response was calculated as an average of two acid responses recorded just prior to peptide application. Averaged responses recorded in the presence of APHC3 were normalized to averaged control responses. Those are presented as the mean ± s.d. (*n* = 4). The inhibition of capsaicin-induced currents by APHC3 was statistically significant (Student test, *p* < 0.05). (**c**, **d**) Cell responses to the acidification of the bath solution from pH 7.4 to pH 5.5 in control and in the presence of the APHC3 applied at different concentrations as indicated. The experimental traces in (**c**) and in (**d**, upper trace) illustrate representative responses of EGFP/TRPV1-positive cells. The bottom trace in (**d**) represents responses of an EGFP/TRPV1-negative cell assayed simultaneously. (**e**) Acid responses recorded in the presence of APHC3 (90, 160 and 240 nM) normalized to a control response. The data are presented as the mean ± s.d. (*n* = 5–10). The inhibition of acid-induced Ca^2+^ transients by APHC3 was statistically significant at 160 and 240 nM (Student test, *p* < 0.05). In each case, the control response was calculated as an average of the two acid responses recorded just prior to peptide application. The average of the two acid responses recorded after peptide application was calculated and normalized to the control response. Experimental traces in (**c**, **d**) were obtained from three different cells. The changes in bath solution pH (continuous lines) and APHC3 applications (thick straight lines) are shown above the fluorescence traces. Cells were stimulated and rinsed by a rapid replacement of the control bath solution with modified bath solution at pH 5.5 with or without APHC3.

Compared to control non-transfected HEK-293 cells (*n* = 5) (not shown), cells transfected with the pIRES2-EGFP/TRPV1 construct exhibited markedly increased sensitivity to capsaicin and low pH, the stimuli known to activate TRPV1 (Figure 2). Given strong correlation between EGFP and TRPV1 expression, solely cells exhibiting high enough green fluorescence were patch clamped, and in some cases, stable recordings were achieved (*n* = 31). Although 100 nM capsaicin elicited strong inward currents in all examined TRPV1-positive cells (Figure 2a), capsaicin responsivity declined with time in most cases (not shown). This phenomenon prevented from conclusive interpretation of long-lasting recordings required for assaying АРНС3 effects on TRPV1 gating. Nevertheless, in four experiments, TRPV1-associated currents were firm enough to suggest the nearly 25% inhibition of TRPV1 currents by 300 nМ АРНС3 (Figure 2a,b).

When TRPV1-positive cells were assayed using Ca^2+^ imaging, 100 nM capsaicin elicited marked Ca^2+^ responses. However, at a serial and prolonged capsaicin application, Ca^2+^ responses were subjected to conspicuous rundown (not shown). Thus, capsaicin appeared to be an unsuitable agonist for examining effects of APHC3 on TRPV1 activity. Unlike capsaicin responses, short acidification of a bath solution could elicit robust Ca^2+^ transients in TRPV1-positive cells throughout 30–60 min recordings (Figure 2c,d).

Importantly, acid stimuli subtly or negligibly affected intracellular Ca^2+^ in control non-transfected HEK-293 cells (*n* = 11) (not shown) as well as in transfected cells that exhibited undetectable EGFP fluorescence, that is, insignificant TRPV1 expression (Figure 2d, bottom trace). Therefore, we studied effects of APHC3 using acid stimuli to activate TRPV1. At the same pH 5.5, the magnitude of Ca^2+^ transients elicited by acid stimuli varied greatly from cell to cell, presumably due to different levels of TRPV1 expression. This inference is supported by the finding that acid responsiveness was in strong correlation with a level of EGFP fluorescence (not shown). The acid responses of TRPV1-positive cells were assayed in the presence of APHC3 applied at different concentrations. The fraction of cells that was capable of generating multiple stable responses to the same acid stimulus was low (~14%) as the response was liable to rundown. Therefore, APHC3 effects on a given cell were only studied at the particular concentration of 90, 160, or 240 nM. Typically, cells were stimulated by 2–3 applications of the acid solution in control, in the presence of APHC3, and after washout of the polypeptide (Figure 2c). Only those cells that generated strong and stable responses in control conditions and after rinse of the polypeptide were used for the statistical analysis. Among the used polypeptide doses, APHC3 exerted statistically significant inhibition of acid responses at the concentrations of 240 nM (by 77% ± 14%) (10 cells) and 160 nM (by 15% ± 9%) (*n* = 7), while 90 nM APHC3 was insignificantly effective (*n* = 5) (Figure 2c–e). The inhibitory effects of APHC3 were almost completely reversible (Figure 2a,c,d).

The residues responsible for APHC’s activity are not determined. The four amino acid substitutions that distinguish APHC1 from APHC3 resulted in significant changes in the ability of these polypeptides to modulate TRPV1 activation. APHC1 inhibited ~32% of capsaicin-induced currents at more than 200 nM [14], while APHC3 had a lower inhibitory effect (25%) even at higher concentrations more than 300 nM. For low pH-induced TRPV1 receptor currents, we were unable to observe any effect of APHC1, whereas APHC3 significantly inhibited pH 5.5 induced currents (approximately 80% at 240 nM). These differences in pharmacology were expressed further in the *in vivo* results.

### 2.2. *In Vivo* Effects of APHC1 and APHC3 in Pain Models

**Figure 3:**
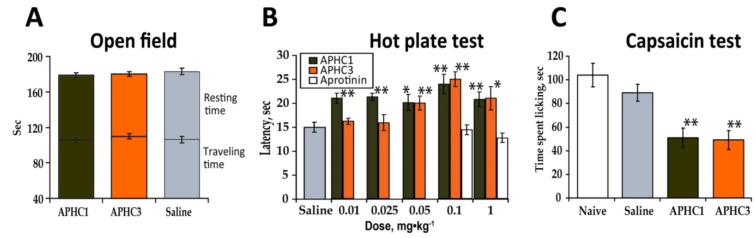
APHC1 and APHC3 did not influence normal mouse behavior but did change the behavioral response to thermal stimuli and capsaicin. (**a**) APHC1 and APHC3 did not significantly alter normal mouse behavior in the open field test at a 0.1 mg/kg dose (*n* = 9 for each group). (**b**) Dose dependent inhibition of thermal nociception by APHC1/3 (*n* = 9 for each group). (**c**) 0.1 mg/kg doses of APHC1 and APHC3 significantly reduced the behavioral response to capsaicin (*n* = 9 for each group). The results are presented as the mean ± s.e.; *—*p* < 0.05, **—*p* < 0.01 *versus* saline group (ANOVA followed by Tukey’s test).

Intensive studies of TRPV1 antagonists and knockout mice showed that this receptor plays a considerable role in many biological processes such as the perception of thermal stimuli, the development of inflammation, inflammatory thermal hyperalgesia and thermoregulation [4]. We conducted multiple *in vivo* experiments to assess the biological effects of APHC1 and APHC3, acting via modulation of TRPV1, on temperature sensation and inflammation. Possible negative effects of investigated components on the central nervous system were first excluded. We evaluated APHC1/3 in open-field locomotor activity tests as compounds capable of reducing locomotor activity could misrepresent results of other behavioral tests (particularly in pain models). We used one polypeptide dose (0.1 mg/kg), which was equal to the most effective polypeptide dose, in following *in vivo* experiments. There was no significant effect detected for either polypeptide at a dose of 0.1 mg/kg i.v. (Figure 3a). Locomotion tests revealed similar travelled distance and rearing compared with the saline control group. Thus, efficacy of polypeptides APHC1/3 in pain models did not resulted from locomotor impairment or sedation.

#### 2.2.1. Hot Plate Test

The hot plate test is a most simple for TRPV1 inhibitor characterization since TRPV1 play significant role in temperature sensation. Effect of APHC1 and APHC3 on thermal nociception at dose 0.1 mg/kg after intramuscular, intraperitoneal and intravenous administration was measured before [15,16]. Each polypeptide significantly prolonged paw withdrawal latency at hot plate after intravenous administration. Here we assessed changes in thermal nociception to a noxious thermal stimuli elicited by different doses of APHC1 and APHC3 to determine minimal effective dose. Intravenous administration of APHC1/3 significantly increased paw withdrawal latency in a hot plate test. APHC1 was shown to be more potent because it produced antinociception even at a 0.01 mg/kg dose (Figure 3b). APHC1 did not prolong paw withdrawal latency at a 0.001 mg/kg dose (data not shown). Both polypeptides reached maximal effect at 0.1 mg/kg. Potent serine protease inhibitor aprotinine with the same structural fold as APHC1 and APHC3 did not change behavioral response to thermal stimuli at doses 0.1 and 1 mg/kg.

This observation of APHC1 and APHC3 reduction of the behavioral response to thermal stimuli, prolonging hot plate latency corresponds well with data on the behavioral output resulting from *in vivo* TRPV1 blockage or disruption of the *trpv1* gene [1]. Also it is important that this effect is not a result of their ability to partially inhibit serine protease since the most potent serine protease inhibitor aprotinine did not change behavioral response to thermal stimuli (Figure 3b).

#### 2.2.2. Capsaicin Test

Because capsaicin is a selective agonist of the TRPV1 receptor, we tested the efficacy of APHC1 and APHC3 as TRPV1 receptor antagonists using the capsaicin-induced pain model. Intraplantar injection of capsaicin evokes pain-related behavior such as licking and shaking the injected paw. TRPV1 knockout mice are insensitive to the capsaicin test and most antagonists that block capsaicin-induced currents *in vitro* also block capsaicin induced pain-related behavior in mice [1]. Intravenous administration of APHC1/3 (0.1 mg/kg) 15 min before capsaicin injection significantly reduced pain-related behavior. Both polypeptides showed an equipotent efficacy in ~44% of response (Figure 3c).

#### 2.2.3. Formalin Test

The formalin test is a complex *in vivo* model of pain where TRPV1’s function is more integrative than perceptive. In the mouse, intraplantar injections of formalin produce a biphasic behavioral reaction. The molecular mechanisms underlying formalin-induced pain are based on tissue damage, non-specific activation of nociceptors and direct activation of TRPA1 receptors (measured in phase I) that drive the local neurogenic inflammation and CNS sensitization (measured in phase 2) [25,26]. APHC1 and APHC3 were tested on TRPA1 for inhibitory/activation effects. They showed neither agonistic nor antagonistic activity to this receptor as well to TRPV3, ASIC3, and p2X3 receptors at concentrations of up to 2 μM in oocyte electrophysiology experiments or agonist-induced fluorescence readout assays (data not shown).

**Figure 4:**
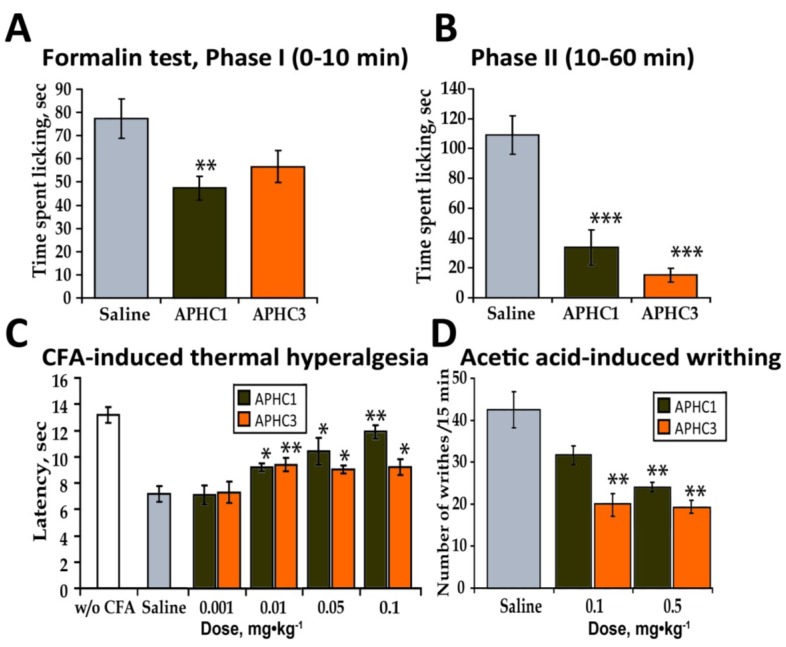
*In vivo* efficacy of APHC1 and APHC3 in inflammatory pain models. (**a**) APHC1 (0.1 mg/kg) significantly attenuated the first phase of the formalin test (*n* = 9 for each group). (**b**) Both polypeptides (0.1 mg/kg) significantly inhibited the second phase of the formalin test (*n* = 9 for each group). (**c**) Dose dependent inhibition of CFA-induced thermal hyperalgesia by APHC1 and APHC3 (*n* = 9 for each group). (**d**) APHC3 (0.1 mg/kg) significantly reduced the number of writhes in the acetic acid-induced writhing test (*n* = 9 for each group). The results are presented as the mean ± s.e.; ***—*p* < 0.001, **—*p* < 0.01, *—*p* < 0.05, *versus* saline group (ANOVA followed by Tukey’s test).

Administration of APHC1 (0.1 mg/kg, i.v.) significantly attenuated the pain-related behavior in the first phase of formalin test (39% of response) and more potently inhibited the second phase (70%). In contrast, APHC3 injection at the same dose (0.1 mg/kg) almost completely inhibited (86%) the second phase of the response but did not affect the first phase of the response (Figure 4a,b). When the dose of the polypeptides was decreased to 0.01 mg/kg no significant influence on the formalin-induced response was found.

An analgesic effect of a TRPV1 antagonist in formalin test was reported previously [27] and we could expect APHC1 and APHC3 to be efficient in amelioration of formalin-induced pain. It is interesting that at the same 0.1 mg/kg dose tested the two polypeptides produce dissimilar effects. APHC1 attenuated both phase 1 and phase 2 of the formalin nocifensive response, but although APHC3 had no significant effect on phase 1, it almost completely blocked phase 2. A possible explanation for this could be that APHC3 inhibits the response by blocking the pH mediated or modulated activation of TRPV1 that could occur during inflammation, whereas APHC1 blocks TRPV1 independently of pH modulation and is more efficient during the activation of nociceptors that occurs in phase 1 of the formalin test. Moreover since TRPA1 is co-expressed with TRPV1 in sensory neurons and their direct interaction has been suggested [28], APHC1 could affect TRPA1 activation via modulation of TRPV1.

#### 2.2.4. CFA-Induced Hyperalgesia

CFA-induced thermal hyperalgesia depends on TRPV1 activation; this was shown in TRPV1-deficient mice and using TRPV1 antagonists [29]. CFA-induced thermal hyperalgesia is a complex process where different inflammatory pathways acting via several mechanisms lower the TRPV1 temperature threshold and influence thermal sensitivity [30]. CFA-treated mice developed thermal hyperalgesia in the injected paw, this manifested as a decrease in the latency to paw withdrawal in response to thermal stimuli (Figure 4c). Both APHC1 and APHC3 significantly reduced thermal hyperalgesia by i.v. administration in doses 0.1-0.01 mg/kg. The APHC1 effect was larger (~80% of reversal) and was clearly dose-dependent. APHC3 had significantly lower efficacy (34% of reversal).

#### 2.2.5. Abdominal Constriction Test of Visceral Pain (Acetic Acid-Induced Writhing)

TRPV1 can be activated by low pH stimuli and is therefore involved in pain caused by acidification, which can be measured in the pain behavior model of acetic acid-induced writhing [27,31]. Intraperitoneal administration of acetic acid provokes a very stereotyped behavior in the mouse. The constriction response is considered the definitive measure of visceral pain intensity [26]. Animals were pretreated with polypeptides 15 min before acetic acid injection. APHC3 administration (0.1 and 0.5 mg/kg, i.v.) significantly reduced the number of writhes observed (50% inhibition); APHC1 did not produce a statistically significant effect at 0.1 mg/kg (i.v.) but dose 0.5 mg/kg (i.v.) significantly reduced number of writhes observed (Figure 4d).

Usually, molecules that inhibit [27] or potentiate [12] pH-induced TRPV1 currents *in vitro* produce similar effects *in vivo*—they significantly reduce the number of writhes after acetic acid injection. This correspondence was confirmed by our results. APHC3 significantly reduced the number of writhes; APHC1 did not significantly inhibit the response to acetic acid at dose 0.1 mg/kg.

### 2.3. APHC1 and APHC3 Decrease the Core Body Temperature

TRPV1 participates in thermoregulation, and almost all its known agonists and antagonists change core body temperature. Selective TRPV1 agonists, such as capsaicin, cause a significant decrease in body temperature. The influence of antagonists on core body temperature is more complex [13,32]. Antagonists that interact with the intracellular capsaicin-binding pocket of TRPV1 elicit a hyperthermic effect if they are able to inhibit pH-induced TRPV1 currents [10,12]. Antagonists that potentiate the pH-induced activation of TRPV1 either decrease or do not change body temperature [12,32]. To date, the best explanation of this hyperthermic response, which occurs when an antagonist is administered, is that the antagonist’s ability to inhibit the constantly activated abdominal TRPV1 receptors provokes a cold defense response [8,33]. However, the factors that keep the receptor in an activated state have not yet been identified. Modulation by pH was proposed as one of the more probable factor [13].

**Figure 5:**
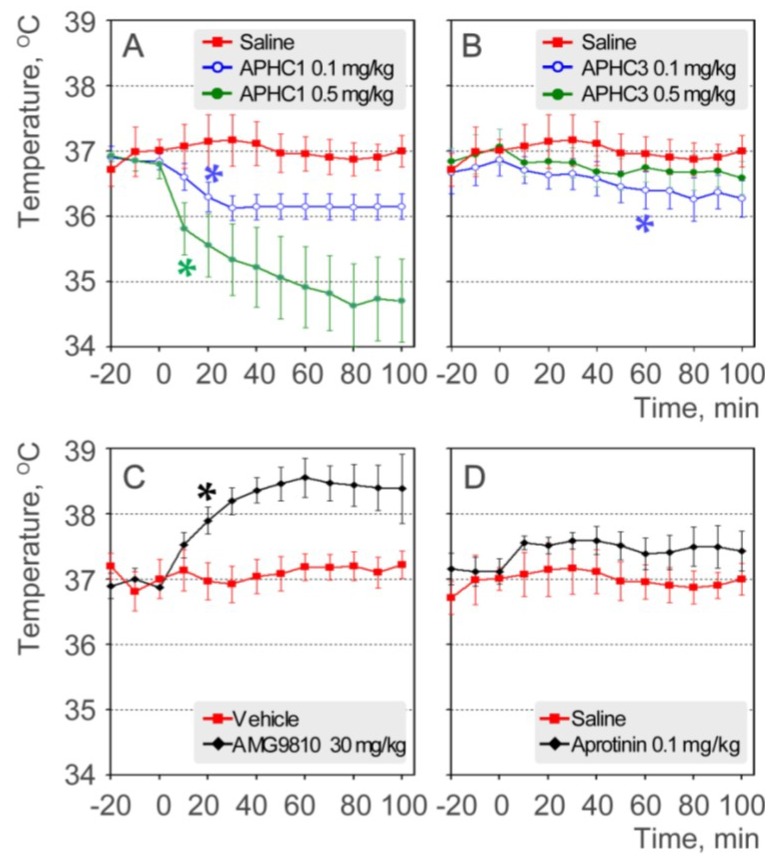
Effects on core body temperature (rectal measuring). (**a**) APHC1 (0.1 and 0.5 mg/kg) and saline (*n* = 7 for each group); (**b**) APHC3 (0.1 and 0.5 mg/kg) and saline (*n* = 7 for each group); (**c**) AMG9810 (30 mg/kg) and vehicle (10% DMSO) (*n* = 7 for each group); (**d**) aprotinin (0.1 mg/kg) and saline (*n* = 7 for each group). The results are presented as the mean ± s.e.; *—*p* < 0.05 *versus* saline group (ANOVA followed by Tukey’s test).

Admittedly, hyperthermia is considered a critical side effect of TRPV1 antagonists, so we assessed the effect of the polypeptides on body temperature. Polypeptides were administered intravenously at doses 0.1 mg/kg and 0.5 mg/kg, and rectal temperature was monitored (Figure 5a,b). An antagonist AMG9810 produced hyperthermic effect was measured as a control (Figure 5c) and aprotinin was used as a control of serine protease inhibitor (Figure 5d).

Injections of saline or the vehicle used for AMG9810 (10% DMSO in saline) did not significantly change the core body temperature of the mice. Administration of AMG9810 (an all modes antagonist of TRPV1) produced a significant increase of 1.6 °C in body temperature, as previously reported [8]. The serine protease inhibitor aprotinin, a polypeptide, caused an increase in core body temperature (0.4–0.5 °C) that was not statistically significant when compared to saline group. In contrast to AMG9810 and aprotinin, both APHC1 and APHC3 at dose 0.1 mg/kg showed a hypothermic effect on the core body temperature of mice. APHC1 produced a rapid decrease in body temperature, −0.8 °C in the 30 min after administration, and the body temperature stayed at this level throughout the remaining data acquisition time. APHC3 caused a slow decrease in body temperature, reaching a change of −0.6 °C 60 min after administration.

APHC1 at dose 0.5 mg/kg produced a more significant decrease in body temperature, −2.1 °C at the end of data acquisition time. APHC3 at dose 0.5 mg/kg produced some hypothermic effect (−0.4 °C) but effect was not statistically significant.

APHC1 significantly decreased core body temperature in both tested doses. APHC3 produced modest hypothermic effect despite it efficiently inhibiting pH-induced TRPV1 currents. Therefore, we can conclude that in case of these peptides the ability to inhibit the pH activation of TRPV1 does not play a significant role in effect on thermoregulation.

Many different sensitized states of TRPV1 are predicted for organisms, both under normal conditions and in pathology. Currently, only a few of these conditions can be modeled *in vitro* because they may be induced by a combination of different factors, such as regulation by multiple protein kinase pathways, endogenous lipids, and extracellular environment (pH, temperature, osmolarity). We hypothesize that antagonists targeted to the capsaicin-binding pocket can efficiently inhibit tonically activated abdominal receptors, while APHC1/APHC3 cannot. Moreover, we can speculate that these peptides (especially APHC1) could potentiate abdominal TRPV1 since they significantly decrease core body temperature. Hypothermic effect of APHC1 and Aphc3 was not the result of their ability to inhibit serine protease since the most potent serine protease inhibitor aprotinine did not change or modestly increase core body temperature (Figure 5d). The ability to decrease core body temperature could be a useful approach to obtain therapeutic hypothermia with additional analgesic (anti-nociceptive/anti-inflammatory effect) properties, as proposed for TRPV1 agonists [34].

## 3. Experimental Section

### 3.1. Production of Recombinant Polypeptides

APHC1 and APHC3 were produced as described previously [14,16,35]. Briefly, DNAs encoding the polypeptides were constructed from synthetic oligonucleotides using PCR and cloned into the expression vector pET32b+ (Merck KGaA, Darmstadt, Germany). *Escherichia coli* BL21(DE3) cells expressing TRX fusions of APHC1 or APHC3 were cultured at 25 °C for 12–14 h, harvested, resuspended in start buffer for metal-affinity chromatography (300 mM NaCl, 50 mM Tris-HCl, pH 7.5), ultrasonicated, and centrifuged to remove all insoluble particles. Fusion proteins were purified using a TALON Superflow Metal Affinity Resin (Clontech, Mountain View, CA, USA). HCl up to 0.5 M and CNBr with a molar ratio to protein of 600:1 were added to the fusion protein solution in the metal-affinity chromatography elution buffer [35]. Cleavage was performed overnight (18 h) at room temperature in the dark. Reactions were terminated by drying the sample under a vacuum. The recombinant APHC1/3 polypeptides were purified on a reverse-phase column Jupiter C_4_ (Phenomenex, Torrance, CA, USA) 250 × 10 mm. The purity of target polypeptides was verified by MALDI-TOF mass-spectrometry.

### 3.2. Cell Culture and Transient Transfection

HEK-293 cells were cultured in Dulbecco’s modified Eagle’s medium (DMEM) (Life Technologies, Grand Island, NY, USA) containing 10% (vol/vol) fetal bovine serum (Thermo Fisher Scientific, Palm Beach, FL, USA), glutamine (1%) and the antibiotic gentamicin (100 μg/mL) (Life Technologies, Grand Island, NY, USA). The pIRES2-EGFP plasmid harboring rat TRPV1 cDNA was transiently transfected into HEK-293 cells by replacing the growth medium in each well with a transfection mixture, containing 1.6 μg of DNA and 4 μL of Lipofectamine (Life Technologies, Grand Island, NY, USA) in 1 mL of serum-free DMEM. After incubating for 6 h, the transfection mixture was replaced with normal culture medium. Cells were assayed within 48–96 h after transfection.

### 3.3. Electrophysiology

Ion currents were recorded using an Axopatch 200A amplifier, with a DigiData 1322A interface, and pClamp8 software (Molecular Devices, Downingtown, PA, USA). External solutions were delivered by a gravity-driven perfusion system at a rate of 0.1 mL/s. Generally, the whole-cell patch clamp approach was used with recording pipettes containing (mM): 140 CsCl, 1 MgATP, 10 BAPTA, 10 HEPES-CsOH (pH 7.2). The basic bath solution included the following (mM): 140 NaCl, 5 KCl, 1 MgCl_2_, 1 CaCl_2_, 10 HEPES-NaOH (pH 7.4).

### 3.4. Single Cell Ca^2+^ Imaging

HEK-293 cells transfected with the pIRES2-EGFP/TRPV1 plasmid were incubated in a bath solution (in mM: 130 NaCl, 10 NaHCO_3_, 5 KCl, 1 MgCl_2_, 1 CaCl_2_, 10 HEPES-NaOH (pH 7.4), 5 glucose) containing 5 μM Fura-2AM and 0.02% Pluronic (both from Life Technologies, Grand Island, NY, USA) for 25 min at room temperature (23–25 °C). Cells were then rinsed twice with the dye-free bath solution and subjected to 20 min incubation for complete Fura-2AM de-esterification. Next, loaded cells were plated onto a coverslip coated with Cell-Tak (BD Biosciences, Franklin Lakes, NJ, USA) inside an attached ellipsoidal resin chamber (150 μL volume) filled with the bath solution. Cells were stimulated by whole chamber perfusion allowing for the complete change of a chamber solution for 2 s. For acid stimulation, HEPES in the bath solution was substituted for MES, and pH was adjusted to 5.5. Fura-2 fluorescence was excited at both 340 nm and 380 nm using two computer-controlled light emitting diodes (LEDs) (Luxion, Irvine, CA, USA). The Fura-2 emission was recorded at 510 ± 40 nm using a Luca R EMCCD camera (Andor Technology, Belfast, UK) attached to an inverted microscope Axiovert 100S equipped with a Plan-Neofluar 40×/0.75 objective (Carl Zeiss, Oberkochen, Germany). Intracellular Ca^2+^ was evaluated by the ratio of F340/F380, where F340 and F380 are intensities of Fura-2 emission at the excitation of 340 nm and 380 nm, respectively. EGFP fluorescence was recorded at 525 ± 40 nm and excited using a LED (Luxion, Irvine, CA, USA) emitting at 480 nm. All recordings were performed at room temperature.

### 3.5. Animals Experiments

All experiments were performed in accordance with the recommendations in the “Principles of Laboratory Animal Care” and after approval by the Animal Care and Use Committee of the Branch of the IBCh RAS (Pushchino, Russia). Adult male CD-1 mice (Animal Breeding Facility Branch of Shemyakin-Ovchinnikov Institute of Bioorganic Chemistry, Russian Academy of Sciences, Pushchino, Russia) weighing 20–25 g were used. Animals were allowed to acclimate in the laboratory for at least five days. Mice were housed at room temperature (23 ± 2 °C) on a 12 h light–dark cycle with food and water available *ad libitum*. Polypeptides or vehicle was administered intravenously 15 min before testing.

The significance of the data was determined by analysis of variance (ANOVA) followed by Tukey’s test. Data are presented as mean ± S.E.

#### 3.5.1. Open-Field Activity in Mice

Open-field activity was measured using a system that counts interruptions in a set of photo beams (OPTO—VARIMEX (Columbus Instruments, Columbus, OH, USA) and ATM3 Auto System using Auto-Track Version 4.2 software). Spontaneous locomotor activity was recorded for 3 min.

#### 3.5.2. Hot-Plate Test

Sensitivity to a thermal stimulus was determined by hind paw withdrawal or licking latency using a Hot-Plate Analgesia Meter (Columbus Instruments, Columbus, OH, USA) at 55 °C.

#### 3.5.3. Capsaicin-Induced Acute Pain

Intraplantar injection of capsaicin (3 µg/10 µL in 10% ethanol/90% saline) was used to elicit capsaicin-induced acute pain [36]. Immediately after the capsaicin injection, mice were placed inside glass cylinders for observation. Intraplantar injection of capsaicin evoked licking and shaking of the injected paw in mice. The duration of episodes of licking and paw shaking were recorded.

#### 3.5.4. Formalin Test

Formalin (10 μL 2% in saline) was injected intradermally into the left hind paw. The left hind paw was observed for 60 min to determine the duration of paw flinching. This test produces a distinct biphasic response [37]. The total duration of paw flinching during the early phase (0–10 min) and late phase (10.01–60 min) were summed.

#### 3.5.5. Complete Freund’s Adjuvant–Induced Thermal Hyperalgesia

Complete Freund’s adjuvant suspended in an oil/saline (1:1) emulsion, was injected into the plantar surface of the left hind-paw of mice (20 μL/paw). Control mice received 20 μL of saline (i.pl.). Injected paw withdrawal latencies in response to thermal stimulation (53 °C) were measured 24 h after CFA injection.

#### 3.5.6. Acetic Acid-Induced Writhing (Abdominal Constriction Test of Visceral Pain)

Separate groups of mice were injected with 0.6% acetic acid in saline (10 mL∙kg^−1^ intraperitoneally (i.p.)). Mice were immediately placed inside transparent glass cylinders, and the number of writhes was recorded for 15 min.

#### 3.5.7. Body Temperature Measurements

The MLT1404 Rectal Temperature Probe and Chart Power Lab (ADInstruments Inc., Colorado Springs, CO, USA) was used to record body temperature. During recording, the animals were placed in a MLA5018 Rodent Restrainer (ADInstruments Inc., Colorado Springs, CO, USA). The adaptation period was 20 min. Core body temperature was recorded every minute for 20 min before and 100 min after test compound administration. AMG9810 was dissolved in DMSO and was injected intraperitoneally.

## 4. Conclusions

This study provides evidence that the partial inhibition of TRPV1 *in vivo* could be more beneficial than its complete inhibition. Polypeptide modulators of TRPV1 showed moderate efficacy *in vitro* (25%–30% block of capsaicin induced current and in the case of APHC3 ~80% block of pH-induced currents). Nevertheless, both APHC1 and APHC3 proved to have significant antinociceptive and analgesic activity *in vivo* at low doses (0.01–0.1 mg/kg). Despite incomplete inhibition of the capsaicin-induced response, APHC1 and APHC3 significantly reduced the pain-related response both in tests directly associated with TRPV1 functions (capsaicin, noxious thermal stimuli, thermal hyperalgesia) and in general models of pain (formalin test, acetic acid writhing). In contrast to the majority of TRPV1 antagonists, which provoke marked hyperthermia *in vivo*, APHC1 and APHC3 caused a moderate decrease in core body temperature. Therefore, the polypeptides APHC1 and APHC3 could be referred to as a new class of TRPV1 modulators that produce a significant analgesic effect without hyperthermia.
